# Osteocytes and Bone Metastasis

**DOI:** 10.3389/fendo.2020.567844

**Published:** 2020-10-14

**Authors:** Manuel A. Riquelme, Eduardo R. Cardenas, Jean X. Jiang

**Affiliations:** Department of Biochemistry and Structural Biology, University of Texas Health Science Center, San Antonio, TX, United States

**Keywords:** osteocyte, bone, cancer, metastasis, microenvironment

## Abstract

Bone is the most frequent site of breast cancer and prostate cancer metastasis, and one of the most common sites of metastasis for many solid tumors. Once cancer cells colonize in the bone, it imposes a major clinical challenge for the treatment of the disease, and fatality rates increase drastically. Bone, the largest organ in the body, provides a fertile microenvironment enriched with nutrients, growth factors and hormones, a generous reward for cancer cells. Dependent on cancer type, cancer cells can cause osteoblastic (bone forming) or osteolytic lesions to promote the net resorption and/or release of growth factors from the bone extracellular matrix. These processes activate a “vicious cycle”, leading to disruption of bone integrity and promoting cancer cell growth and migration. Cancer cells influence the bone microenvironment favoring their colonization and growth. In order to metastasize to the bone, cancer cells must first migrate from the site of origin, and once established within the bone, they must overcome the dormant inducing effects of resident cells. If successful, cancer cells can then colonize and continually disrupt bone homeostasis that is primarily maintained by osteocytes, the most abundant bone cell type. For example, it has been shown that exercise induces osteocytes to release anabolic factors that inhibit osteoclast resorptive activity, promote dormancy and the release of anti-cancer factors that inhibit breast cancer cell metastasis. In this review, we will summarize recent research findings and provide mechanistic insights related to the role of osteocytes in osteolytic metastasis.

## Introduction: Cancer Bone Metastasis

The bone is a mineralized tissue highly regulated to adapt and meet the diverse needs of the host relative to physical demand, hormones, metabolic state, and environmental stimulation. Bone remodeling involves three major bone cell types; osteoblasts (bone forming cells) and osteoclasts (bone resorbing cells) that function in maintaining the structural balance, and the osteocytes that function in bone remodeling in response to environmental and mechanical signals and stimuli (1). The osteocyte, which is the most abundant cell type (~95%) in the bone, is the primary cell responsible for bone remodeling and homeostasis. Embedded inside the bone mineral matrix, osteocytes are connected and able to sense and respond coordinately to environmental cues, such as hormones, physical stress, and mechanical loading and unloading. These properties allow osteocytes to modulate the bone microenvironment by promoting the release of factors that regulate bone formation or resorption with respect to demands. Disease and aging can disrupt bone homeostasis, create structural defects, and alter the bone macro- and microenvironment, ultimately leading to cancer cells colonizing within the tissue (2). The bone, along with the liver and lung, is one of the most frequent sites of cancer metastasis (2). Bone metastasis is an unfortunate outcome of many solid tumors, typically breast, lung, prostate, thyroid, renal carcinoma, melanoma, gastrointestinal tumors, and head and neck cancers (2, 3). Tumors that originate in the bone represent a small fraction of diagnosed cancers. Originating from cells found in bone tissue of osteosarcomas, these cancers are of transformed osteoblastic lineage, and occur most often in adolescents (4, 5). Other cancers, such as multiple myeloma arise from the bone marrow, but do not come from mesenchymal lineage. Approximately 80% of bone lesions and tumors originate in the bone marrow as multiple myeloma (3, 6, 7). The process of other cancers metastasizing to the bone is as complicated as one would expect considering that it is not one single cancer type that favors bone metastasis. This article primarily focuses on cancer bone metastasis from cancer not originating from the bone. Bone metastasis greatly affects the quality of life of patients, causing complications, such as pain, nerve root or spine cord compression, vertebral or peripheral fractures, hypercalcemia, and bone marrow infiltration that lead to cytopenia (3, 8).

The reasons why tumor cells metastasize to the bone are poorly understood. Bone tissue provides an ideal microenvironment for metastatic tumor cells. Bone marrow endothelium, adipocytes, and the immune response all participate in maintaining bone homeostasis in ways that are only partially understood. Although solid tumor metastasis to the bone is common, not all cancers preferably metastasize to the bone. Thus, disseminated tumor cells homing to the bone may be a targeted, and/or the microenvironment found in the bone, including cellular, hormonal or otherwise is not suitable for the growth of certain cancer types (8, 9). Interestingly, highly vascularized bone containing red bone marrow and cancellous bone (*e.g.* pelvis and long bones) are common sites of metastasis (rarely hand and foot bones) (3). It has been shown that primary tumors from near and distal regions of the body organize and make ready premetastatic niches. For instance, myeloid cells can be recruited from the bone marrow by tumor-derived exosomes that release a plethora of soluble factors, including, proteins, enzymes, and small nucleic acids, which are capable of homing in circulating tumor cells to the newly forming metastatic niche (10).

*α*v*β*3 integrins (acting as cell surface adhesion receptors) have been found to play a key role in mediating the metastatic MDA-MB-231 and Chines Hamster Ovary tumor cells into the bone (11, 12). Metastatic cancer cells are attracted and retained in the bone marrow through the sensing and signaling of chemokines, for example, the C-X-C motif chemokine ligand 12 (CXCL12), which is expressed in bone marrow stromal cells, attracts tumor cells overexpressing the C-X-C chemokine receptor type 4 (CXCR4) (13). In another study by Cox et al., lysyl oxidase (LOX) was identified in hypoxic ER-negative breast tumor cells to play a key role in preparing the bone metastatic niche. LOX induces osteoclastogenesis independent of RANKL, disrupts bone homeostasis, ultimately leading to the formation of premetastatic bone lesions (14). Moreover, high LOX activity has been clinically associated with increased collagen cross-linking, fibrosis, and elevated risk of cancer metastasis (15). LOX, secreted by primary tumor cells, is responsible for catalyzing the cross-linking of both collagen and elastin, which increases matrix stiffness, alignment, and total ECM volume. The increase of ECM stiffness facilitates the activation of integrins and augments Rho-generated cytoskeletal tension promoting focal adhesion formation and cell motility (14, 15).

Adaptive immune cells also play a role in setting up the bone metastatic niche. Immune-competent mice orthotopically injected with metastatic 4T1 breast cancer cells are shown to have increased osteoclastogenesis; this induces the pre-metastatic osteolytic niche required for colony formation. It is further shown that the primary tumor environment promotes the differentiation of helper T cells (CD4^+^), and the tumor-specific Th17 cells expressing RANKL, which stimulates osteoclast activation and induces osteolytic bone lesions, ultimately promoting breast cancer colonization in the bone (16).

**Figure 1:**
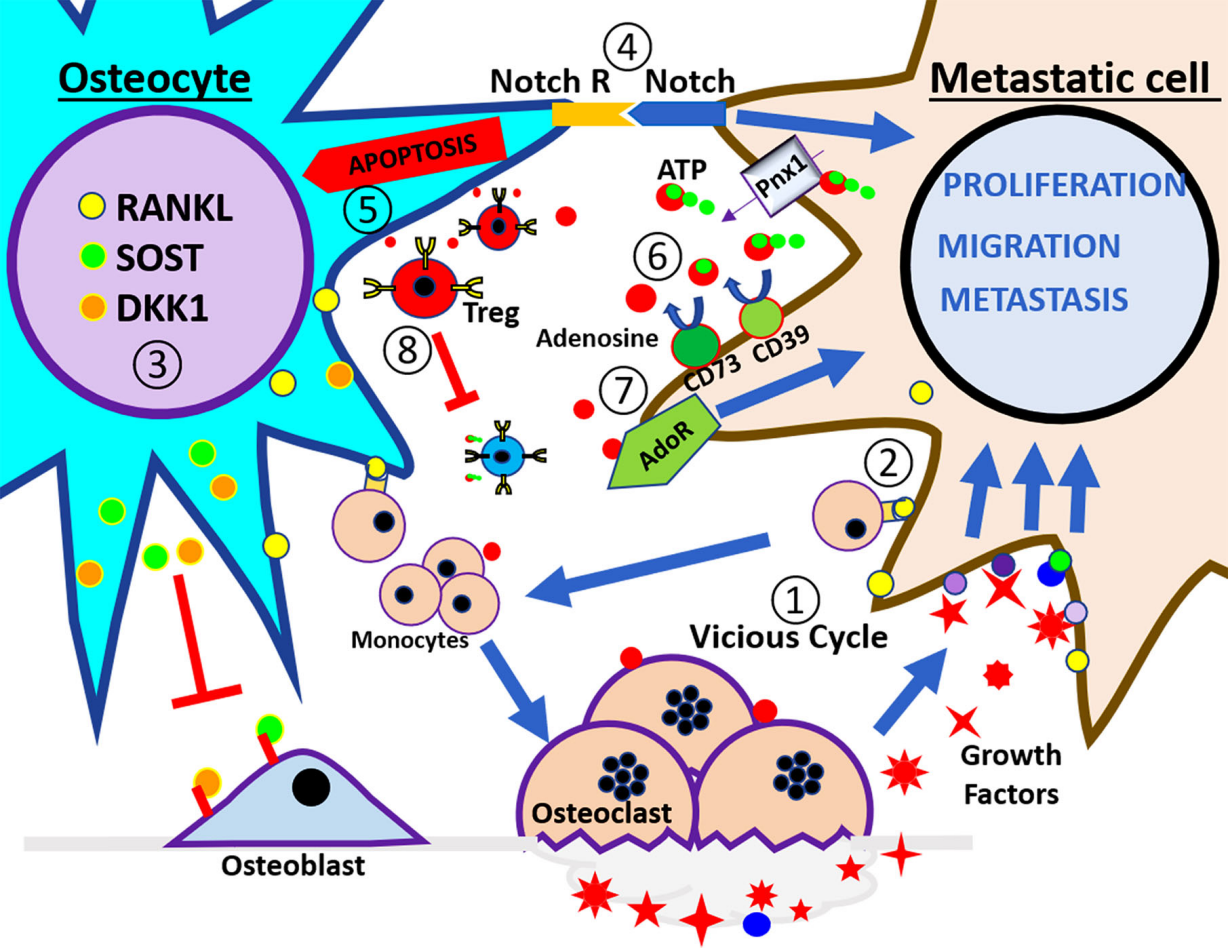
Schematic illustration of tumor microenvironment in the bone. At the left side of the panel is the osteocyte and the right side is the breast cancer cell. These cells interact in a bone catabolic environment. The numbers indicate the steps of events that may happen during breast metastasis described in the review. ① The vicious cycle; cancer cells interact with monocytes to increase the osteoclast number and activity in order to release growth factors embedded in the bone. ② RANKL expressed by osteocytes and cancer cells increases the recruitment of monocytes and stimulates the osteoclast differentiation. ③ Under this condition osteocytes increase the release of Sost and DKK1 that inhibit osteoblast activity and increase the expression of RANKL. ④ Notch signaling pathway induces apoptosis in the osteocytes and increases the proliferation of cancer cells. ⑤ Osteocytes apoptosis signals promote osteoclasts for bone resorption. ⑥ eATP is hydrolyzed to adenosine through CD39/CD73 enzyme activation, and generated adenosine activates adenosine receptor in cancer cells, leading to increased proliferation, migration, and metastasis. ⑦ Extracellular adenosine increases osteoclast activity, ⑧ also promotes Treg activity, and increases immune tolerance.

The seemingly self-perpetuating metastatic growth to bone has been described as a ‘vicious cycle’. In an intricate process inside the bone, tumor cells secrete osteoclastogenic factors (*e.g.*, IL-1, IL-6, IL-11, PDGF, MIP1*α*, TNF, M-CFS, RANKL, and PTHrP) that help stimulate the recruitment and activity of osteoclast, key players in the formation of osteolytic lesions (17). This process disrupts bone homeostasis and induces the release of growth factors, including, activin, transforming growth factor *β* (TGF*β*), fibroblast growth factor (FGF), and platelet-derived growth factor (PDGF) from the bone mineral matrix. In turn, these released factors promote tumor cell growth and increase further bone resorption (**Figure 1**, step ①) (18). This feedback loop, or ‘vicious cycle’, increases the incidence of metastatic lesions in the bone and eventually leads to related ailments, *e.g.* bone fractures, and high levels of blood calcium (hypercalcemia).

As mentioned, an important player in the vicious cycle is osteoclasts, large bone resorbing multinucleated cells originating from the fusion of bone marrow-derived monocytes/macrophages. Activated osteoclasts adhere to bone surfaces, forming an acting ring that covers a space in which bone demineralizing enzymes and proteases are secreted. Key players in osteoclast differentiation include adenosine nucleotides, receptor activator of nuclear factor κ-B ligand (RANKL), macrophage colony-stimulating factor (M-CSF), and other molecules (19), which are principally generated from nearby osteoblasts, osteocytes, and immune cells (20). Osteoclast generation and activation is achieved directly, or indirectly by RANKL production by neighboring cells, or by bone trophic tumor cells. These activities are eventually used by tumor derived cells to create the bone niche, leading to further osteoclastogenesis and bone resorption. The mechanistic comprehension of bone turnover in tumor growth has led to the clinical use of osteoclast inhibiting bisphosphonates, and Denosumab (anti-RANKL antibody) in patients with bone metastasis, and has become the standard of care to improve quality of life by limiting bone turnover (**Figure 1**, step ②) (21). In addition to molecules directly involved in bone resorption, other factors involved in bone resorption include interleukins-6 and 11 (22), parathyroid hormone-related peptide (PTHrP) (23, 24), soluble intercellular adhesion molecule 1 (ICAM-1) (25), Wnt molecules (26, 27), macrophage-stimulating protein (MSP) (28), and extracellular adenosine (**Figure 1**, step ③) (29).

Although crucial to bone metastasis and creating the metastatic niche, osteoclasts are not the only cell type to participate in bone metastasis, and osteoblasts also play a vital role. Osteoblasts participate in matrix mineralization, which provides strength (hardness) to the bone (30). Osteoblasts are derived from skeletal bone marrow stromal cells that differentiate into preosteoblasts and secrete numerous factors, including RANKL that directly impact osteoclastogenesis (**Figure 1**, step ③) (30). These cells eventually differentiate into mature osteoblasts, which secrete the mineral matrix proteins and mineralize bone (30). Some osteoblasts may become bone lining cells or become embedded in lacunae where they differentiate into fully mature mechanosensing osteocytes (1, 30). While studies show osteoclasts can induce tumor proliferation by releasing growth factors stored within the mineral matrix, osteoblast activity has been associated with tumor cell growth and tumor cell dormancy. Preosteoblasts and osteoblasts express tumor-promoting osteoprotegerin (OPG) (31), hepatocyte growth factor (HGF), and secrete connective tissue growth factor (CTGF) and TGF*β* (22). Furthermore, osteoblasts express IL-6, which increases osteoclastogenesis, and has been shown to drive proliferation of multiple myeloma plasma cells (24, 32).

**Figure 2:**
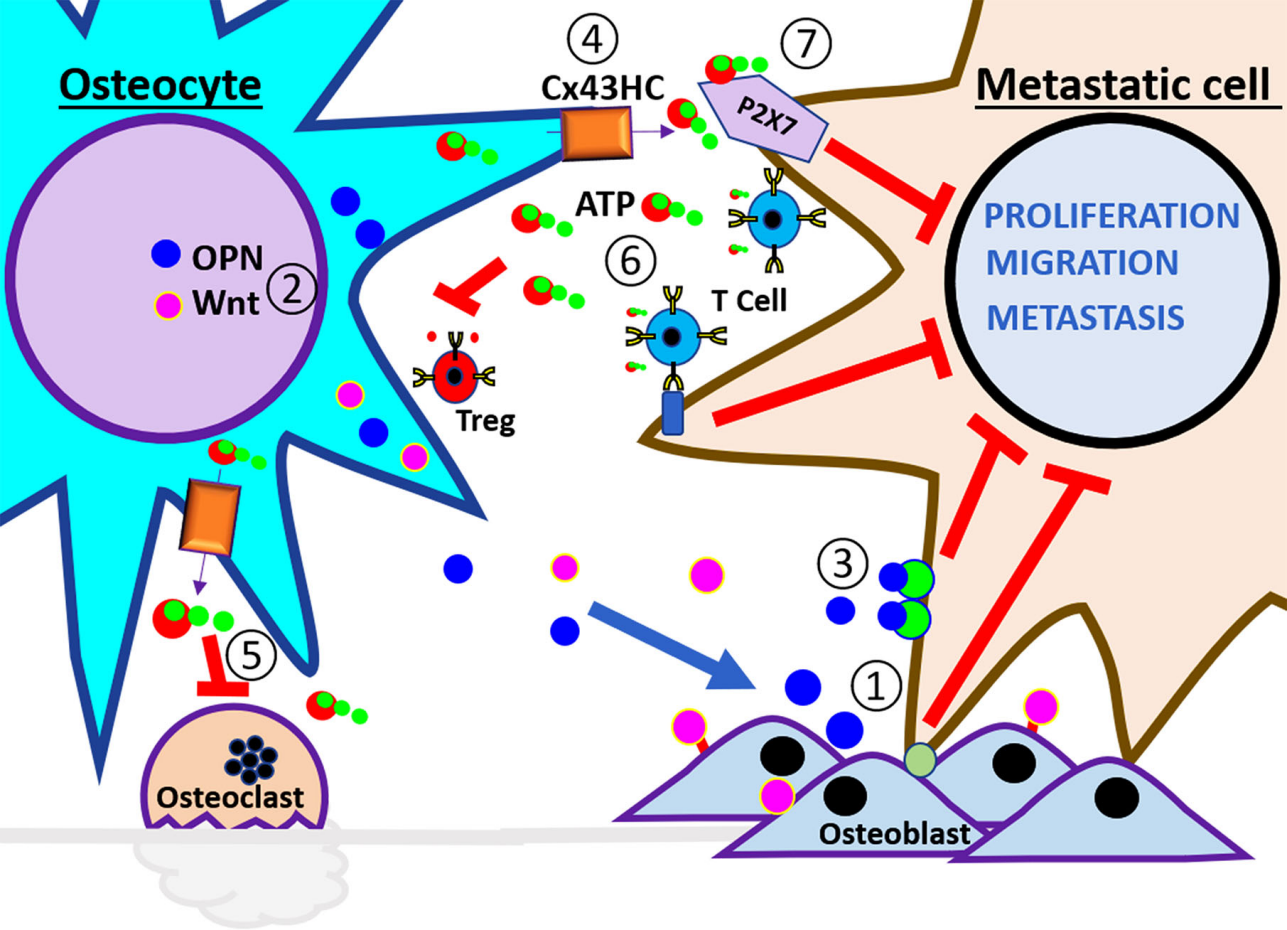
Schematic illustration of anti-tumor microenvironment in the bone. At the left side of the panel are the osteocytes and the right side are the breast cancer cells. These cells interact in a healthy bone environment. The numbers indicate the steps of events that may happen during breast metastasis described in the review. ① The interaction of metastatic cells with osteoblast promotes dormancy. ② Physiological level of mechanical loading stimulates osteocyte release of anabolic factors, Wnt, and OPN, to increase osteoblast differentiation, activity, and bone strength. ③ High concentration of OPN reduces EMT in metastatic cells. ④ Mechanical loading increases opening of Cx43 hemichannels and the release of ATP. ⑤ eATP inhibits osteoclast activity and ⑥ also inhibits Treg formation and stimulates immune surveillance. ⑦ eATP activates P2X receptor and reduces the proliferation, migration, and metastatic potential of cancer cells.

Tumor–osteoblast interactions have been shown to be critical in establishing bone metastasis (33, 34). Circulating (prostate) metastatic cells have been shown to have an affinity for the bone endosteal surface where they interact with osteoblasts through annexin2/annexin2 receptor interactions (33, 34). These micro-metastases are formed in regions of new bone formation, where differentiating and actively mineralizing osteoblasts are located. Furthermore, osteoblast and breast tumor interaction is shown to require adherent junction formation for tumor cell proliferation (9), supporting the notion that factors produced during osteogenesis promote cancer proliferation. Disseminated cancer cells must also compete for the endosteal surface of the bone, a niche occupied by non-proliferating long-term hematopoietic stem cells (LT-HSCs) (**Figure 2**, step ①). The mechanisms of cell cycle arrest of breast cancer cells once established in the endosteal niche are likely the same mechanisms that induce the non-proliferating status of long-term resident hematopoietic stem cells, unfortunately, only to later escape dormancy and proliferate (35, 36). In one study using a 3D co-culture model (osteoblast/breast cancer), it was identified that the addition of bone remodeling cytokines, tumor necrosis factor (TNF)-α and interleukin (IL)-1*β* and tumor necrosis factor resulted in increased proliferation in the breast cancer cell line MDA-MB-231 BMRS1 (37). The inhibition of TNF-α and IL-1*β* downstream targets, cycloxygenase (COX) and PGE2 receptors, resulted in decreased cancer cell proliferation (37). Moreover, osteoblasts and osteocytes also secrete leukemia inhibitory factor (LIF), and the activation of LIF receptors present in breast cancer cells is shown to maintain them in a dormant state. Loss of LIFR resulted in decreased expression of genes associated with cell dormancy. LIFR knockdown increased cancer cell migration and invasion, proliferation, and osteoclastogenesis. Interestingly, overexpression of PTHrP also decreased LIFR signaling. (38).

An early study by Kobayashi et al. found bone stromal cells induced dormancy by the release of bone morphogenic protein 7 (BMP7) and activation of prostate BMP receptor 2. BMP7-treated prostate cancer cells resulted in activated p38 MAPK, increased expression of p21 and the metastasis suppressor gene, NDRG1 (N-myc downstreamregulated gene 1) (39). Key studies have given further insight into the mechanisms in which osteoblasts may induce dormancy in disseminated tumor cells (40, 41). In another study, osteoblast conditioned media increased cellular quiescence of prostate cancer cells. TGF-*β*2 and growth differentiation factor (GDF)10 were identified as osteoblast secretory factors that induced quiescence in several prostate cancer cell lines. The binding of these factors to the TGF-*β*RIII receptor expressed in prostate cancer cell lines activated (phosphorylation at Thr180/Tyr182) p38 mitogen activated protein kinase (MAPK). Activated p38-MAPK phosphorylation of downstream target retinoblastoma protein (Rb) resulted in the inhibition of cancer cell-cycle progression (40). In another study of prostate cancer metastasis to the bone, Yumoto et al. identified osteoblast-derived ligand growth arrest specific 6 (GAS6) and the tumoral tyrosine kinase receptor Axl as required for the TGF-*β*2-induced response towards prostate cancer cell dormancy (41). Multiple myeloma cells have also been shown to be affected by the bone microenvironment. These cells can occupy the endosteal niche, remain dormant, and escape therapies that largely target dividing cells. The interaction of multiple myeloma cells with cells of osteoblastic lineage along the endosteal bone surface was associated with single, non-dividing tumor cells. Interestingly, dormant myeloma cells that were insensitive to melphalan, a chemotherapeutic agent, could be reactivated upon osteoclast activation with the soluble form of RANKL (42).

The bone is home to the hematopoietic system, and it integrates an assortment of systemic physiological signals. Bone homeostasis is affected directly or indirectly by many pathological conditions, including diabetes, gastrointestinal diseases, physical stress, *etc*. (43–48). One example is the increased risk of cancer and tumor growth under inflammatory conditions (32, 49) or the propensity of cells metastasizing to fractures sites (50). Similar correlations have been associated with surgical procedures (51–53), which intrinsically induce trauma, inflammation, and an increase in innate immune cells needed for tissue repair. These responses have been shown to promote conditions conducive to metastatic growth at non-surgical sites (51, 53).

Bone, like other organs, changes with age, which includes an accumulation of senescent cells, such as, osteoblasts, and resident bone cells harboring genetic mutations (54–56). The loss of bone density with age is well known. Equally concerning is how the accumulated damage to cells and their genetic makeup, caused by environmental toxins, or byproducts of cellular respiration, can result in an environment primed for tumor growth and metastasis (57). How does an increase in senescent bone cells affect certain cancers metastasizing to the bone, or dormant cells in the bone being released from dormancy? As discussed here and shown in numerous studies, senescent osteoblasts can promote osteoclastogenesis, which leads to increased metastasis, dissemination, and metastatic growth of cancer in the bone. This supports and explains the possible mechanisms in which elder cancer patients in remission with dormant cancer cells in the bone often relapse. A possible underlying reason is that cancer cells can take residence in the bone through the contribution of senescent cells accumulated over time (42, 58). An important detail to keep in mind is that most experimental studies have not used aged animal models (59, 60). Studies delving into how age affects metastasis are in dire need.

Bone impacts metastasis in unexpected and sometimes complex manners. Mechanical loading inhibits secondary growth and osteolytic capability of metastatic tumors in nude mice by modulating osteoblastic/osteoclastic activities and communication between osteocytes and tumor cells (61). Osteocytic release of osteopontin (OPN) (**Figure 2**, step ②), a secreted phosphoprotein with a high avidity to bone mineral matrix, has been reported to induce activators of the EMT process (62). Interestingly, lower OPN levels (0.1 to 0.5 μg/ml) induce EMT markers. Low levels of mechanical loading (1 N) are shown to increase the expression and secretion of OPN (Fan, 2020 #82). The increase in OPN in turn inhibits the expression of TGF-*β* in osteocytes, increases the adhesion of tumoral cells, thus possibly inhibiting growth and migration by anchoring tumor cells at the primary site. This bone microenvironmental condition is pro-mesenchymal to epithelial transition (MET) reducing the aggressiveness and allowing the settlement of secondary tumor (**Figure 2**, step ③) (63). Similar intensities of mechanical loading inhibiting tumor growth in nude mice have been previously reported (61). As studies have shown, high loading intensity of the bone enhances breast cancer cell malignancy; therefore, the extent of mechanical loading should be carefully monitored (63). Taken together, studies indicate osteocytes are an important player in providing the ‘soil’ for bone metastasis/progression as well as associated skeletal diseases (27, 64–66).

## Intricate Function of Osteocytes in Bone Homeostasis and Cancer Bone Metastasis

**Figure 3:**
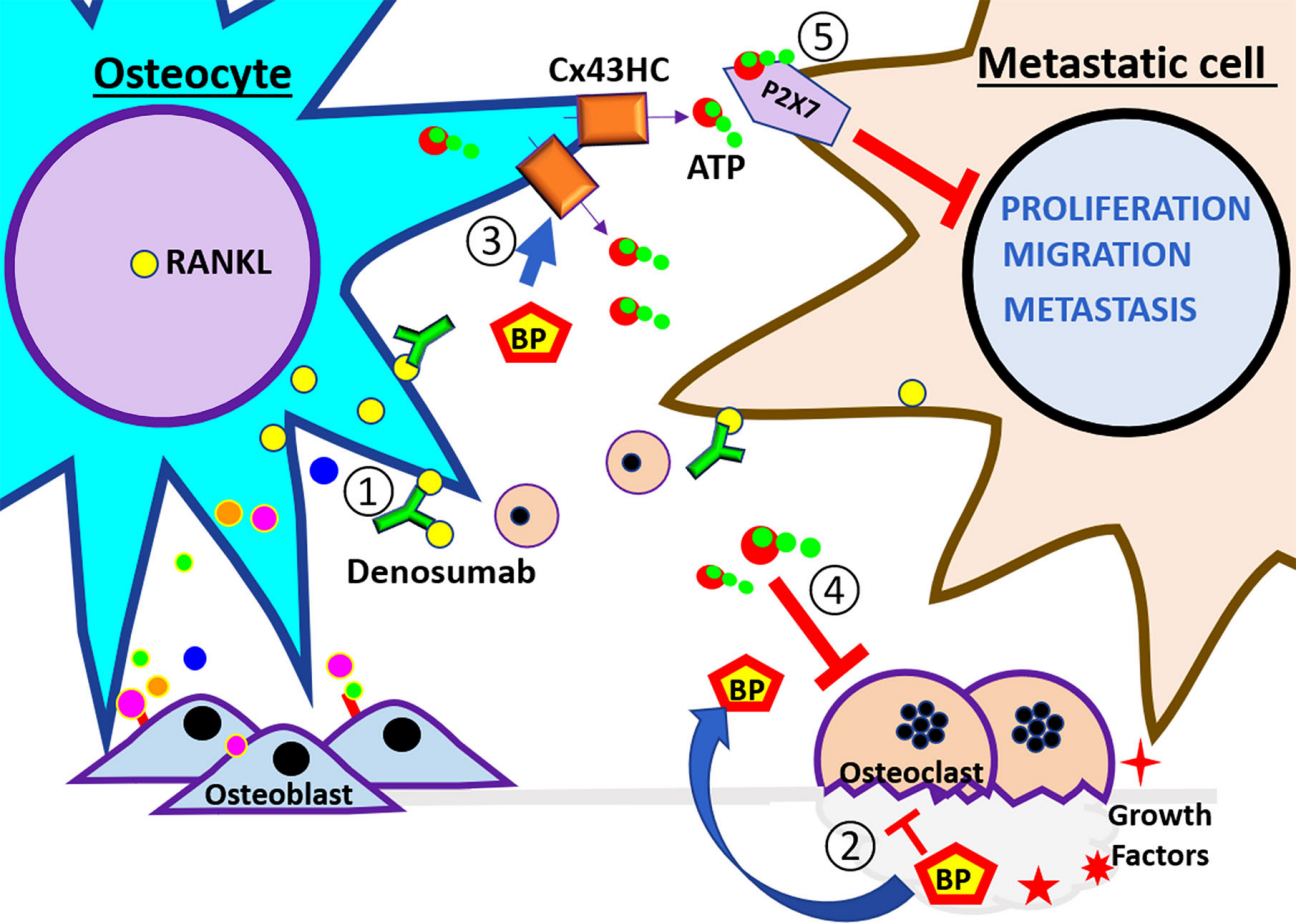
Schematic illustration of therapeutic agents used to treat bone metastasis. At the left side of the panel are the osteocytes and the right side are the breast cancer cells. These cells are subjected to the therapeutic treatment under the bone environment. The numbers indicate the steps of events that may happen during breast metastasis described in the review. ① Denosumab, a RANKL neutralizing antibody, binds RankL expressed by osteocytes and cancer cells, and inhibits the recruitment of monocytes and osteoclast differentiation. ② Bisphosphonates (BPs) inhibit osteoclast activity. Also, ③ BP increases osteocyte survival and induces opening of Cx43 hemichannels and the release of ATP. ④ eATP inhibits osteoclast activity. ⑤ eATP activates P2X receptor and reduces the proliferation, migration, and metastatic potential of cancer cells. The effects of the pharmacological agents reduce the activity of the vicious cycle.

The extensive lacuna–canaliculi network allows osteocytes to directly communicate with one another. It also allows for osteocytes to respond to local and distant signals, including mechanical (bone stress) or biological (paracrine and endocrine) (1, 67). Osteocytes control bone remodeling through regulation of bone-forming and bone-destroying cells. During bone demineralization, osteocytes decrease osteoblast differentiation and function through secreted factors, including the Wnt signaling antagonists sclerostin and Dickkopf Wnt signaling pathway inhibitor 1 (DKK1) (67). Osteocytes are the main sclerostin producer in the bone, and this protein inhibits the association of Wnt ligands with their receptor in osteocytes and osteoblast. Therefore, the key bone formation inhibitor, sclerostin, has been given much attention as a targeted therapeutic approach for low bone density (66, 68). Bone RANKL is primarily produced by osteocytes (**Figure 1**, step ③). RANKL promotes monocyte differentiation into bone resorbing osteoclasts. Neutralization of RANKL with the antibody Denosumab is currently in use to reduce fracture incidence in low bone density, bone metastasis, and rare bone cancers (**Figure 3**, step ①) (69, 70). The Denosumab Clinical trial (ABCSG-18) showed that postmenopausal breast cancer patients under aromatase inhibitor treatment had a significant latency of apparition of bone fracture with Denosumab as an adjuvant (71). This was also true in metastatic breast cancers and new primary malignancies.

In contrast, in a clinical trial (D-CARE study) of patients with stage II/III breast cancer, Denosumab treatment did not improve bone metastasis free survival. Denosumab did increase the incidence of osteonecrosis of the jaw (5 *versus <*1%) and hypocalcaemia (7 *versus* 4%) in comparison with placebo (72). Although the above mentioned trials do not corroborate Denosumab having a beneficial effect on survival, the results obtained from these two trials have a significant difference. The D-care trial patient profile was of early stage high risk breast cancer patients, while the ABCSC-18 study focused on early stage low risk breast cancer patients. It is also important to note the trials were conducted with different Denosumab schedules and endpoints (70).

As mentioned above, bones inevitably age and undergo numerous changes, including bone loss, osteocyte apoptosis, and increased oxidative stress. Osteocyte apoptosis is a key stimulus that triggers bone resorption (**Figure 1**, step ⑤) (73, 74). The slowed production of sex hormones that comes with aging promotes osteoclast activity (75, 76), osteocyte apoptosis (73), elevated oxidative stress (76, 77), and a reduction in osteoblast function (78). Thus, reduction in sex hormones culminates in bone fragility and bone loss. This bone destructive environment is further enhanced by a decline in immune surveillance and increased fat formation; this disturbs the balance of critical osteoclastogenic proteins, RANKL, and OPG, towards bone destruction (79). The AZURE phase 3 clinical trial for post-menopausal women was designed to study the effects of adjuvant zoledronic acid treatment in early high risk breast cancer patients. The AZURE trial showed that with treatment, incidence of bone metastasis was reduced. Critical to our understanding, this benefit was restricted to postmenopausal women and those under ovarian suppression treatments with hormone-receptor-positive breast cancer. This observation highlights how critical it is to understand the differences between old and young bones in metastasis (80, 81).

Emerging studies indicate how osteocytes could have a positive impact on tumor growth, motility, and survival, a scenario leading to poor outcomes in cancer patients. The interaction between prostate cancer cells and osteocytes induces the osteocytic production and release growth-derived factor 15 (GDF15) promoting prostate cancer cell proliferation, migration, and invasion of prostate cell in the bone (82). MLO-Y4 osteocytes stimulated by hydrostatic pressure, similar to what is observed in bone metastasis, increased the viability of prostate, breast, and lung cancer cell lines. Hydrostatic pressure also improved motile and invasive capacity through increased expression of chemokine (C-C motif) ligand 5 (CCL5, also known as RANTES) and MMPs (83). Likewise, high intensity shear stress increased survival and migration in the MDA-MBA-231 breast cancer cell line (63, 84). However, these studies also show how intensity of load imparts opposing effects on survival and migration. This underscores the importance of osteocytes in the modulation of the bone microenvironment with regard to tumor progression particularly with respect to the relationship between the magnitude of mechanical stimulation and breast cancer cell apoptosis or migration (84).

Increased osteocyte apoptosis within lytic bone lesions has been found in patients with multiple myeloma (85). During the progression of multiple myeloma, osteocytes directly interact with multiple myeloma cells, which stimulate osteocytes to produce sclerostin and RANKL. This results in the recruitment of osteoclast precursors and a reduction of Wnt signaling, leading to the inhibition of osteoblast differentiation (**Figure 1**, step ③). Concomitantly, cell to cell interactions reduce osteocyte viability due to apoptosis triggered Notch signaling and sustained by multiple myeloma derived TNFα. Furthermore, Notch signaling interaction increases the proliferation of multiple myeloma by increasing cyclin D1 RNA levels and accelerating cell proliferation (**Figure 1**, step ④) (27). This highlights how osteocytes play a constant integrative role of endocrine, paracrine, and mechanical signals, and the output of those signals results in bone formation or resorption responses. The complexity of such integration makes it particularly difficult to predict the impact of osteocytes on cancer cells metastasized to the bone.

## Protective Roles of Osteocytes Against Cancer Bone Metastasis

The skeleton is a dynamic organ that responds to physical stress by promoting bone remodeling, which includes the addition and removal of bone. Although several resident bone cells are involved in mechanosensing, osteocytes are regarded as the major mechanosensory cell within the bone (67, 86). The long dendritic processes of osteocytes form gap junction channels composed primarily by gap junction proteins (connexins). These gap junction networks connect not only neighboring osteocytes, but also cells on the bone surface, including osteoblasts and osteoclasts (1, 67). The mechanosensing osteocytes form connexin 43 (Cx43) hemichannels (half a gap junction channel) which allows for the communication between the internal environment of the cell and its extracellular environment. Gap junction channels are involved in the global regulation and fine tuning of bone formation and resorption as it was evidenced by the altered levels of serum remodeling markers N-terminal propeptide of type I procollagen and C-terminal telopeptide of type I collagen, respectively (87). Osteocyte hemichannels likely play a predominant role in its response to mechanical stimulation; given that bone and osteocytes are constantly subjected to mechanical stimuli as a result of physical movement, gravity, and blood circulation. This is evident by the major impact that Cx43 hemichannels have on the expression of OPG and RANKL, and osteocyte viability, which are essential for bone integrity and longevity (87, 88). Concordantly, the anti-apoptotic effect of bisphosphonates on osteoblasts and osteocytes has been shown to be through regulation of Cx43 hemichannels (**Figure 3**, step ③) (89). Bisphosphonates are the gold standard for therapy for bone diseases in cancer patients (6, 80) as they inhibit osteoclast activity and prevent bone loss induced by cancer cells, thus reducing fracture risk (21, 28). *In vivo*, osteocyte Cx43 hemichannel activity is an important mediator of the growth inhibitory effects of bisphosphonates in breast cancer (65). *In vitro* and *in vivo* studies by our group further underscore the key role of Cx43 in mediating the tumor inhibitory effects of bisphosphonates. Bisphosphonate conditioned media from osteocytes inhibited breast cancer cell growth (MDA-MB-231), migration, and invasion. These effects were abrogated by treating with Cx43 hemichannel specific blocking antibody (65). Moreover, mice with impaired Cx43 gap junctions and hemichannels showed significantly increased tumor burden and a reduced effect of bisphosphonates on tumor growth compared to mice with impaired Cx43 gap junction channel function or wild type (65). This implies Cx43 hemichannels in osteocytes are responsive to bisphosphonates, thus making Cx43 a promising novel drug target for the treatment of breast cancer metastasis to the bone. In a previous study, we have shown that these effects are mediated by adenosine triphosphate (ATP) released by osteocyte Cx43 hemichannel opening (**Figure 2**, steps ④ and ⑦) (**Figure 3**, steps ③ and ④) (90). These important findings highlight that more work is needed to determine exactly how osteocytes impact various cancers metastatic potential, and if these cells can be targeted to stop bone metastasis.

## ATP Release by Osteocytes, a Key Component for the Hostile Microenvironment for Cancer

As a response to tissue damage and cellular stress, cells, including osteocytes, secrete/release ATP to the extracellular space (**Figure 2**, step ④) (65, 91, 92). The intracellular concentration of ATP is ~3–10 mM, and the extracellular ATP (eATP) is about 10 nM. The big difference between intracellular and extracellular ATP is from active ATP degradation through ectonucleotidases in the extracellular compartment (93). The presence of eATP has been shown to inhibit the growth of pancreatic, colon, prostate, breast, liver, ovarian, colorectal, esophageal, melanoma, and leukemia (94). Intravenous ATP used in clinical trials of patients with pre-terminal lung cancer, showed an increase in survival rate and had a beneficial effect on weight and muscle strength (95–97). Multiple studies show an anticancer action of eATP or eATP analogs by binding to P2 purinergic receptors (90, 98). eATP is rapidly degraded to adenosine, a well-known tumorigenic factor (92, 93, 99). Additionally, eATP or P2 receptor agonist decreases osteoclast activity and bone resorption (**Figure 2**, step ⑤) (100), reduces the number of T regulatory lymphocytes (Tregs) (101), and prolongs the activity of T lymphocytes (**Figure 2**, step ⑥) (102). However, ATP metabolites through P1 purinergic receptor activation also mediate pro-tumorigenic effects in prostate and breast cancer cells (90, 103). This suggests that ATP and/or ATP metabolite balance plays a key role in the tumor microenvironment. The solid tumor microenvironment is usually hypoxic and/or inflammatory, and the extracellular concentration of nucleotides (ATP/adenosine) is higher in comparison to normal tissue (92, 103, 104). In this hypoxic/inflammatory microenvironment, adenosine promotes cancer cell migration and chemotaxis in breast cancer and melanoma cells, along with an increase in osteoclastic activity and bone resorption (**Figure 1**, ⑥ and ⑦) (29). This microenvironment also results in poor immune surveillance with high lymphocytic tolerance (**Figure 1**, steps ⑥and ⑧) (92, 105, 106). The main pathway leading to high extracellular adenosine levels is the hydrolysis of eATP by a family of enzymes known as ectonucleotidases, such as CD39 and CD73, which hydrolyze ATP and ADP to AMP, and AMP further to adenosine (**Figure 1**, step ⑥) (93, 105). CD73 has been associated with a pro-metastatic phenotype in breast cancer, and CD73 knockdown leads to suppression of breast cancer cell growth, migration, and invasion both *in vivo* and *in vitro* (105). Therefore, we must practice caution since the function of eATP on tumorigenesis could largely depend on the activity of ecto-ATPases in the tissue.

Adenosine and ATP bind to specific purinergic receptors at the cell surface, which are divided into P1 receptors, with adenosine as the main ligand, and P2 receptors, with ATP and ADP as the main agonists. P1 receptors have four subtypes: A1, A2a, A2b, and A3. There are two major P2 receptor subtypes, seven P2X, and eight P2Y subtypes (92, 107). The presence of P2Y subtypes has been shown to play important roles in cell survival under mechanical stress, although the role of specific P2X subtypes remains unclear. It has been suggested that autocrine activation of breast cancer P2X7 receptors participates in the activation of cell proliferation, cancer cell process elongation, and further ATP release (99). The tumor microenvironment rich in ATP is shown to either promote or inhibit cell migration, enough to activate multiple P2 receptors, but not enough to induce cell death trough P2X7 activation. The study by Zhou et al. provides clues to clarify the mixed effects of ATP. They showed that the addition of a non-hydrolysable P2X receptor agonist resulted in an inhibitory effect on cancer cell migration and growth. This inhibitory effect was dependent on P2X7 activation (**Figure 2**, step ⑦) (**Figure 3**, step ⑤) (90). A2A receptor activation, on the other hand, resulted in a stimulatory effect on breast cancer cell migration and growth (**Figure 1**, step ⑦) (90). Cancer cell-specific expression of P1 receptor subtypes has been reported. for example, in breast cancer. A2b receptors are absent on ER-positive MCF-7 cells, whereas MDA-MB-231 cells express very high levels of A2b (90). In order to understand and establish strategies to control tumor growth and metastasis, it is very important to evaluate the presence and expression levels of the specific P1 or P2 receptor subtypes in cancer cells. These are likely key factors determining if a cellular response to adenosine nucleotides will be elicited.

## Conclusion and Future Directions

The osteocyte is a key player modulating the bone cancer microenvironment. Metastatic cancer cells have shown the potential to utilize osteocyte signaling by turning the bone microenvironment osteoclastogenic and transforming osteocytes into pro-tumorigenic cells. Moreover, osteocytic overproduction of Wnt inhibitors contributes to the suppression of bone formation. In addition, metastatic cancer cells colonized in the bone reduce osteocyte viability, resulting in reduced cell capacity to maintain bone homeostasis. However, the bone forming ability of osteocytes is related with an anti-resorptive microenvironment. This condition reduces osteocyte apoptosis, enhances Cx43 hemichannel activity, increases bone strength, and reduces osteoclast recruitment and activity. Taken together, these osteoclast activities will inhibit the overall activation of metastatic dormant cells, and tumor growth, and motility. Although we have just started a fascinating journey to understand the functional relationship between cancer and osteocytes, targeting osteocyte signaling pathways and molecular messengers has already shown to have a positive impact on preventing/improving bone pathologies associated with cancers. These results offer encouraging and supportive ideas of a targeted approach on osteocytes that reside in the cancer niche. These studies may offer guidance and a map to develop new therapies for cancers metastasized to bone, or the prevention thereof.

In conclusion, osteocytes play a large role as gate keepers of the bone and bone homeostasis. By having a broader knowledge on how osteocytes influence cancer cells, osteoblasts, and osteoclasts, we may improve and increase pharmacological strategies to help keep the bone healthy and free of cancer. Further work is needed to uncover key events and players that coordinate the communication between cancer cells and bone cells. The goal is to identify target points that can disrupt the initial and key steps of bone metastasis. Osteocytes may prove to be a key ally in combating cancer cell progression in the bone.

## Author Contributions

All authors contributed to the article and approved the submitted version.

## Funding

JJ was supported by the US National Institutes of Health grant CA196214, US Department of Defense grant BC161273, and Welch Foundation grant AQ-1507.

## Conflict of Interest

The authors declare that the research was conducted in the absence of any commercial or financial relationships that could be construed as a potential conflict of interest.
